# Self-reported chemicals exposure, beliefs about disease causation, and risk of breast cancer in the Cape Cod Breast Cancer and Environment Study: a case-control study

**DOI:** 10.1186/1476-069X-9-40

**Published:** 2010-07-20

**Authors:** Ami R Zota, Ann Aschengrau, Ruthann A Rudel, Julia Green Brody

**Affiliations:** 1 Silent Spring Institute, 29 Crafts Street, Newton, MA 02458, USA; 2 Department of Epidemiology, Boston University School of Public Health, Talbot 3E, 715 Albany Street, Boston, MA 02118, USA

## Abstract

**Background:**

Household cleaning and pesticide products may contribute to breast cancer because many contain endocrine disrupting chemicals or mammary gland carcinogens. This population-based case-control study investigated whether use of household cleaners and pesticides increases breast cancer risk.

**Methods:**

Participants were 787 Cape Cod, Massachusetts, women diagnosed with breast cancer between 1988 and 1995 and 721 controls. Telephone interviews asked about product use, beliefs about breast cancer etiology, and established and suspected breast cancer risk factors. To evaluate potential recall bias, we stratified product-use odds ratios by beliefs about whether chemicals and pollutants contribute to breast cancer; we compared these results with odds ratios for family history (which are less subject to recall bias) stratified by beliefs about heredity.

**Results:**

Breast cancer risk increased two-fold in the highest compared with lowest quartile of self-reported combined cleaning product use (Adjusted OR = 2.1, 95% CI: 1.4, 3.3) and combined air freshener use (Adjusted OR = 1.9, 95% CI: 1.2, 3.0). Little association was observed with pesticide use. In stratified analyses, cleaning products odds ratios were more elevated among participants who believed pollutants contribute "a lot" to breast cancer and moved towards the null among the other participants. In comparison, the odds ratio for breast cancer and family history was markedly higher among women who believed that heredity contributes "a lot" (OR = 2.6, 95% CI: 1.9, 3.6) and not elevated among others (OR = 0.7, 95% CI: 0.5, 1.1).

**Conclusions:**

Results of this study suggest that cleaning product use contributes to increased breast cancer risk. However, results also highlight the difficulty of distinguishing in retrospective self-report studies between valid associations and the influence of recall bias. Recall bias may influence higher odds ratios for product use among participants who believed that chemicals and pollutants contribute to breast cancer. Alternatively, the influence of experience on beliefs is another explanation, illustrated by the protective odds ratio for family history among women who do not believe heredity contributes "a lot." Because exposure to chemicals from household cleaning products is a biologically plausible cause of breast cancer and avoidable, associations reported here should be further examined prospectively.

## Background

Pesticides, household cleaners, and air fresheners are of interest in breast cancer research because many contain ingredients that are mammary gland carcinogens in animals [1] or endocrine disrupting compounds (EDCs), including compounds that affect growth of estrogen-sensitive human breast cancer cells [2] or affect mammary gland development [3]. Mammary gland tumors have been observed in animal studies of pesticides such as dichlorvos, captafol, and sulfallate; methylene chloride (in some fabric cleaners); nitrobenzene (soaps, polishes); and perfluorinated compounds (stain-resistant, waterproof coatings) [1,4,5]. Phthalates, alkylphenols, parabens, triclosan, and polycyclic musks used as surfactants, solvents, preservatives, antimicrobials, and fragrances have shown weak estrogenic or anti-androgenic effects in both *in vitro *and *in vivo *tests [4-16]. Pesticides identified as EDCs include dichlorodiphenyl trichloroethane (DDT), chlordane, methoxychlor, atrazine, lindane (lice control), vinclozolin and benomyl (fungicides), and several current use insecticides such as cypermethin [6-13]. When given early in life, atrazine, nonylphenol, perfluorinated compounds, and the plastics monomer bisphenol A influence rat mammary gland development in a way that may affect tumor susceptibility [14-18]. These chemicals are widely used and many have been detected in blood and urine from a representative sample of the US population; concentrations vary over several orders of magnitude [19-26]. In household air and dust and women's urine tested in the Cape Cod Breast Cancer and Environment Study, we detected an average of 26 EDCs per home, including 27 pesticides and a variety of estrogenic phenols from household cleaners [27]. Taken together, the laboratory studies of biological activity and evidence of widespread human exposure suggest that use of products containing mammary gland carcinogens or EDCs may contribute to breast cancer in humans.

No epidemiological studies we know of have reported on the relationship between cleaning product use and breast cancer, and previous breast cancer studies of pesticides have been largely limited to organochlorine compounds [28]. Organochlorine studies have been mostly null, but interpretation is limited because proxies of exposure were measured in blood taken years after the compounds were banned in the US, often in older women and after diagnosis [29]. In a study that avoids these limitations by using archived blood collected from young women in 1959 to 1967, Cohn et al. [30] reported five-fold higher breast cancer risk among women who had the highest residues of DDT and were exposed before they were 14 years old. In addition, the Long Island Breast Cancer Study found 30% higher breast cancer risk among women who reported the highest home pesticide use [31]. Self-reported product use, such as the Long Island measures, has the potential to represent exposure over many years to a wide range of compounds; although retrospective reports may be biased by differential reporting accuracy between cases and controls [32].

To investigate the relationship between use of cleaning and pesticide products and risk of breast cancer, while considering possible recall bias, we conducted a case-control study of breast cancer and self-reported product use on Cape Cod, Massachusetts, in which we also measured beliefs about breast cancer causation, a possible source of recall bias. Cape Cod is a coastal peninsula where breast cancer incidence has been elevated. Annual female breast cancer incidence in 2002 - 2006 was 151.0 per 100,000 (95% CI 142.6 - 159.8) [33]. The pattern of higher incidence in Cape Cod towns than elsewhere in Massachusetts dates to the initiation of the state cancer registry in 1982 [34]. In the Collaborative Breast Cancer Study, risk was elevated among Cape Cod women compared with other Massachusetts participants after controlling for breast cancer risk factors [35]. In the Cape Cod Breast Cancer and Environment Study case-control study, longer years of residence on Cape Cod was associated with higher risk after controlling for established breast cancer risk factors [36].

## Methods

### Study population

Details of the Cape Cod Study have been described previously [37]. Briefly, we conducted a case-control study of invasive breast cancer occurring on Cape Cod in 1988-1995. Cases were female permanent residents of Cape Cod for at least six months before a breast cancer diagnosis reported to the Massachusetts Cancer Registry (MCR). Controls were female permanent Cape Cod residents during the same years, had resided there at least six months, and were frequency matched to cases on decade of birth and vital status. Controls under 65 years of age were selected using random digit dialing; controls over 65 years of age were randomly selected from the Centers for Medicare and Medicaid Services (CMS).

The Cape Cod Study expands on a study of breast cancer and tetrachloroethylene (PCE) in drinking water [38]. Cases diagnosed in 1988-1993 in eight towns and their controls were interviewed in 1997-1998 in the PCE study. Cases diagnosed in 1994-1995 in those eight towns and in 1988-1995 in the remaining seven towns and their controls were interviewed in 1999-2000. Among 1,578 eligible living and deceased cases identified by MCR, 1,165 women (74%) or their proxies participated, 228 (14%) could not be located or contacted, and 185 (12%) refused to participate. Among 1,503 eligible controls, 1,016 (68%) participated.

For the present analysis, we excluded 368 cases and 287 controls who were interviewed by proxy, and 10 cases and eight controls who were missing data for one or more key analytic variables. Given that most women for whom we obtained proxy interviews were deceased, excluded women were older, and, consistent with being older, they were less educated. Within the included or excluded groups, cases and controls did not differ demographically, suggesting no selection bias. Exclusions left 787 cases and 721 controls for pesticide analyses. Cleaning product questions were asked only in 1999-2000 interviews, resulting in 413 cases and 403 controls for whom these data were available.

We obtained permission to use confidential data from MCR, CMS, and hospitals where cases were diagnosed. The Boston University Institutional Review Board and Massachusetts Department of Public Health Human Research Review Committee approved the study protocol. Participants were asked for informed consent at the outset of interviews.

### Interviews

Trained telephone interviewers administered a structured questionnaire on established and hypothesized breast cancer risk factors including family history of breast cancer, menstrual and reproductive history, height, weight, alcohol and tobacco use, physical activity, pharmaceutical hormone use, and education. Information on residential cleaning product and pesticide use was obtained. Participants in 1999-2000 interviews were asked about five categories of cleaning products, including solid and spray air fresheners, surface cleaners, oven cleaners, and mold/mildew products. All participants were asked about use of 10 categories of pesticides in and around their homes, including insecticides, lawn care, herbicides, lice control, insect repellents, and pest control on pets. The 1999-2000 interviews asked about mothballs and treatments for termites and carpenter ants. Participants were first asked if the product was ever used in their home. Participants were then asked to estimate frequency of use using predefined categories. To exclude exposures after diagnosis or index year, participants were asked to report the first and last years of use for pesticides, and use before their diagnosis or index year for cleaning products. At the end of the interview, participants were asked about their beliefs about four factors that may contribute to breast cancer: heredity, diet, chemicals and pollutants in the air or water, and a woman's reproductive or breastfeeding history. Participants were asked whether each contributes to breast cancer "a lot, a little, or not at all." "Don't know" responses were coded. Interview questions can be viewed at http://silentspring.org/cape-cod-breast-cancer-and-environment-study-survey-instruments.

### Statistical analysis

Unconditional logistic regression was used to calculate odds ratios (ORs) and 95% confidence intervals (CIs). The following "core" matching variables and potential confounders were included in adjusted odds ratio analyses based on *a priori *consideration of the research design and well-established breast cancer risk factors: age at diagnosis or index year, education, family history of breast cancer in a first degree female relative, breast cancer diagnosis prior to the current diagnosis or index year, and age at first live or still birth (≥ 30 years of age or nulliparous vs. < 30 years of age). Pesticide analyses were adjusted for study (PCE or Cape study). Missing values for family history for 45 (3%) participants were imputed as "no." The percent missing information on family history did not differ between cases and controls. The following potential confounders were evaluated: mammography use, medical radiation, lactation, hormone replacement therapy, oral contraceptive use, diethylstilbestrol exposure, body mass index, smoking, alcohol consumption, teen and adult physical activity, race, marital status, and religion. None of these variables changed the "core"-adjusted odds ratio estimates by ≥ 10%, so they were not included in final models.

We evaluated ever vs. never use and categorical variables reflecting frequency of use. "Never users" of each product type formed the reference group. If a participant reported ever using a product but the frequency was missing, frequency was imputed as the median for that product. To aggregate "like" exposures, three variables were constructed by summing frequency of use for two types of air fresheners, five types of cleaning products, and eight types of pesticides. Aggregated scores were divided into quartiles based on the distribution of controls. The lowest quartile constituted the reference group. Tests for trends were conducted by modeling ordinal terms for categories of product use or quartiles in the multivariate model.

Because participants' awareness of a hypothesis may bias exposure reporting [39], we evaluated differences in beliefs about disease causation between cases and controls using the chi square test. We evaluated differences in product-use odds ratios by beliefs about whether chemicals/pollutants contribute to breast cancer by 1) including an interaction term for beliefs and product use in the final model and 2) stratifying by beliefs. Beliefs were dichotomized as those who said chemicals/pollutants contribute to breast cancer "a lot" versus "a little," "not at all," or "don't know."

Weiss [40] notes that recall bias is not the only explanation for differences in odds ratios by knowledge or attitudes about a hypothesis; so to aid interpretation of product use results, we conducted a comparison analysis of differences in family history odds ratios by beliefs about whether heredity contributes "a lot" to breast cancer. This comparison is useful, because the accuracy of self-reported family history can be compared with medical records, and the relationship between family history and breast cancer is well-established independent of self-reports. As a sensitivity analysis, we also examined un-stratified and stratified family history odds ratios excluding those subjects who were missing information on family history.

All analyses were conducted in SAS version 9.1 (SAS Institute, Cary, NC). Figures were constructed in R software 2.6.1, (R Foundation for Statistical Computing, Vienna, Austria). Statistical significance was defined by a (two-sided) *P *-value of 0.05 or lower.

## Results

Study participants were predominantly white (98%), 60-80 years of age (60%) with high school or higher education (94%); more cases (25%) than controls (19%) reported a family history of breast cancer. Characteristics of participants are shown in Table 1. Participants in this analysis of product use were demographically similar to characteristics previously reported for all cases and controls, except for being younger and more educated, due to exclusion of proxy interviews [37].

**Table 1:** Characteristics of Cape Cod Breast Cancer and Environment Study participants with completed pesticide use self-reports

	Cases		Controls	
	(N = 787)		(N = 721)	
**Characteristic**	**N**	**%**	**N**	**%**

Age at diagnosis or index year				

< 50	128	16	149	21

50-59	115	15	129	18

60-69	277	35	226	31

70-79	221	28	184	26

≥ 80	46	6	33	5

				

Education				

< High school graduate	36	5	48	7

High school graduate	241	31	226	31

1-3 years college/vocational school	253	32	230	32

College graduate	144	18	122	17

Graduate work/degree	113	14	95	13

				

Family history of breast cancer				

Yes	196	25	135	19

No	591	75	586	81

				

Prior history of breast cancer				

Yes	48	6	46	6

No	739	94	675	94

				

Age at first live or stillbirth				

< 20	171	22	122	17

20-29	104	13	80	11

> = 30	458	58	456	63

Nulliparous	54	7	63	9

				

Menopause status at diagnosis or index year				

Pre-menopause	144	19	194	28

Post-menopause	615	81	505	72

Data for 27 cases and 18 controls were missing for the "Family history of breast cancer" characteristic. Data for 28 cases and 22 controls were missing for the "Menopause status at diagnosis or index year" characteristic.

### Products use

Breast cancer risk increased approximately two-fold in the highest compared with lowest quartile of combined cleaning product use (OR = 2.1, 95% CI: 1.4, 3.3) and combined air freshener use (OR = 1.9, 95% CI: 1.2, 3.0) (Table 2). Ever use of air freshener spray (OR = 1.2, 95% CI: 0.9, 1.8), solid air freshener (OR = 1.7, 95% CI: 1.2, 2.3) or mold/mildew control (OR = 1.7, 95% CI: 1.2, 2.3) was associated with higher risk, with evidence of positive dose response and significant *P*_trend _for solid air freshener and mold/mildew control with bleach. Surface and oven cleaners were not associated with breast cancer risk.

**Table 2:** Adjusted odds ratios for breast cancer and reported cleaning product use, Cape Cod, Massachusetts, 1988-1995

Product category	Cases (No.)	Controls (No.)	Adjusted OR	95% CI	***P ***_**trend**_
Combined cleaning product use					

Quartile 1	91	99	1.0	Reference	

Quartile 2	100	107	1.1	0.8, 1.7	

Quartile 3	112	125	1.1	0.7, 1.7	

Quartile 4	104	70	2.1	1.4, 3.3	0.003

					

Combined air freshener use (sprays and solids)					

Quartile 1	74	77	1.0	Reference	

Quartile 2	113	117	1.1	0.7, 1.7	

Quartile 3	123	138	1.0	0.7, 1.6	

Quartile 4	101	71	1.9	1.2, 3.0	0.02

					

Air freshener spray					

Never use	90	95	1.0	Reference	

Any use	322	308	1.2	0.9, 1.8	

					

< Once a month	83	88	1.1	0.7, 1.7	

Monthly	47	41	1.3	0.8, 2.3	

Weekly	114	110	1.3	0.8, 1.9	

Daily	78	69	1.3	0.8, 2.1	0.15

					

Solid air freshener					

Never use	259	288	1.0	Reference	

Any use	153	115	1.7	1.2, 2.3	

					

< 2 times/year	50	41	1.4	0.9, 2.2	

2-6 times/year	77	58	1.7	1.2, 2.6	

≥ 7 times/year	26	16	2.0	1.0, 4.0	0.001

					

Oven cleaner					

Never use	33	33	1.0	Reference	

Any use	379	370	1.0	0.6, 1.7	

					

< 2 times/year	145	143	1.0	0.6, 1.8	

2-6 times/year	199	196	1.0	0.6, 1.7	

≥ 7 times/year	35	31	1.2	0.6, 2.3	0.80

					

Surface cleaner					

Never use	53	54	1.0	Reference	

Any use	359	348	1.1	0.7, 1.7	

					

< Once a month	61	60	1.0	0.6, 1.6	

Monthly	57	57	1.0	0.6, 1.8	

Weekly	186	171	1.2	0.8, 1.9	

Daily	55	60	1.2	0.7, 2.2	0.22

					

Mold/mildew control					

Never use	296	322	1.0	Reference	

Any use	114	81	1.7	1.2, 2.3	

					

Mold/mildew control with bleach					

Never use	320	334	1.0	Reference	

Any use	90	68	1.5	1.0, 2.1	

					

< Once a month	47	38	1.2	0.8, 2.0	

Monthly	14	11	1.5	0.7, 3.5	

≥ Weekly	29	19	2.0	1.1, 3.8	0.02

Odds ratios are adjusted for age at diagnosis/reference year, birth decade (six categories), previous breast cancer diagnosis, family history of breast cancer, age at first live or still birth (< 30, ≥ 30/nulliparous), education (five categories). "Combined cleaning product use" combines frequency of use across five product categories: air freshener spray, solid air freshener, oven cleaner, surface cleaner, and mold/mildew control with bleach.

Combined use of pesticide products was not associated with risk of breast cancer (Table 3). Odds ratios for individual pesticide types were null or slightly and nonsignificantly elevated, with the exception of insect repellent use (OR = 1.5, 95% CI: 1.0, 2.3 for most frequent insecticide use compared with never use; *P*_trend _= 0.05).

**Table 3:** Adjusted odds ratios for breast cancer and residential pesticide use, Cape Cod, Massachusetts, 1988-1995

Product category	Cases (no.)	Controls (no.)	Adjusted OR	(95% CI)	***P ***_**trend**_
					

Combined pesticide use					

Quartile 1	173	152	1.0	Reference	

Quartile 2	110	99	1.0	0.7, 1.5	

Quartile 3	169	143	1.1	0.8, 1.5	

Quartile 4	153	126	1.1	0.8, 1.6	0.52

					

Insect or bug control					

Never use	161	151	1.0	Reference	

Any use	569	514	1.1	0.9, 1.4	

					

Once or twice	161	155	1.0	0.7, 1.4	

3-10 times	203	188	1.1	0.8, 1.5	

> 10 times	205	171	1.2	0.8, 1.6	0.21

					

Termite or carpenter ant control					

Never use	293	265	1.0	Reference	

Any use	165	161	0.9	0.6,1.2	

					

Once or twice	105	85	1.0	0.7,1.5	

3-10 times	35	49	0.6	0.4,1.0	

> 10 times	25	27	0.8	0.4,1.4	0.11

					

Mosquito control					

Never use	314	312	1.0	Reference	

Any use	91	87	1.0	0.7, 1.5	

					

Once or twice	15	18	0.9	0.5. 1.9	

3-10 times	35	31	1.1	0.7, 1.9	

> 10 times	41	38	1.0	0.6, 1.7	0.79

					

Mothball control					

Never use	73	91	1.0	Reference	

Any use	340	312	1.2	0.8, 1.7	

					

< 5 times	92	90	1.2	0.8, 1.9	

5-10 times	62	73	0.9	0.6, 1.5	

> 10 times	186	149	1.3	0.9, 1.9	0.29

					

					

Lawn care					

Never use	316	286	1.0	Reference	

Any use	408	343	1.1	0.9, 1.3	

					

Once or twice	43	35	1.2	0.7, 1.9	

3-20 times	174	136	1.2	0.9, 1.6	

> 20 times	191	172	1.0	0.7, 1.3	0.88

					

Outdoor and indoor plant care					

Never use	407	359	1.0	Reference	

Any use	334	300	1.0	0.8, 1.2	

					

Once or twice	33	26	1.1	0.6, 1.8	

3-20 times	158	146	1.0	0.7, 1.3	

> 20 times	143	128	1.0	0.7, 1.3	0.71

					

Insect repellent					

Never use	286	271	1.0	Reference	

Any use	482	428	1.2	0.9, 1.5	

					

Rarely	283	263	1.1	0.9, 1.5	

Sometimes	133	115	1.2	0.9, 1.7	

Often/Very often	66	50	1.5	1.0, 2.3	0.05

					

Lice control					

Never use	692	626	1.0	Reference	

Any use	89	83	1.2	0.8, 1.6	

					

Flea collar for pets					

No	257	238	1.0	Reference	

Yes	529	482	1.2	0.9, 1.5	

					

Flea control for pets					

Never use	465	395	1.0	Reference	

Any use	294	286	1.0	0.8, 1.2	

					

Once or twice	43	41	0.9	0.6, 1.5	

3-10 times	101	109	0.9	0.6, 1.2	

> 10 times	150	136	1.1	0.8, 1.4	0.95

Odds ratios are adjusted for age at diagnosis/reference year, birth decade (six categories), previous breast cancer diagnosis, family history of breast cancer, age at first live or still birth (< 30, ≥ 30/nulliparous), education (five categories), study (Cape, PCE). "Combined pesticide use" product category includes frequency data for: insect or bug control, lawn care, outdoor and indoor plant care, insect repellent, flea control on pets. Product use for termite or carpenter ant control, mosquito control, and mothball control not included because they were only assessed in study participants from the 1999-2000 interviews.

### Differences by beliefs about disease causation

Cases and controls differed significantly in beliefs about the role of heredity and of chemicals and pollutants in breast cancer (Table 4). Among controls, 66% said heredity contributes "a lot" compared with 42% of cases (*P *< 0.01); 57% of controls and 60% of cases said "chemicals and pollutants in the air or water" contribute "a lot" (*P *< 0.05).

**Table 4:** Beliefs about the causes of breast cancer by case status, Cape Cod, Massachusetts, 1988-1995

		Cases		Controls		
**How much does ... contribute to breast cancer?**		**No.**	**%**	**No.**	**%**	

Heredity	A lot	331	42	474	66	**

	A little	295	37	163	23	

	Not at all	99	13	36	5	

	Don't know	62	8	48	7	

						

Diet	A lot	217	28	205	28	

	A little	327	42	294	41	

	Not at all	160	20	125	17	

	Don't know	83	11	97	13	

						

Chemicals and pollutants in the air or water	A lot	476	60	412	57	*

	A little	188	24	203	28	

	Not at all	53	7	31	4	

	Don't know	70	9	75	10	

						

Women's reproductive or breast feeding history	A lot	67	9	70	10	

	A little	262	33	261	36	

	Not at all	245	31	225	31	

	Don't know	213	27	165	23	

						

Percentages may not add to 100% because of rounding. Two-sided P value calculated using chi square test; * indicates P < 0.05 and ** indicates P < 0.001.

In stratified analyses, odds ratios for cleaning products were consistently elevated within the group who said chemicals/pollutants contribute "a lot" to breast cancer, but associations moved towards the null in the other participants (Table 5). For example, the odds ratio for the highest quartile of combined cleaning product use was 3.2 (95% CI: 1.8, 5.9) among women who believed chemicals/pollutants contribute "a lot" compared to 1.2 (95% CI: 0.6, 2.6) among others. The interaction was not statistically significant (*P *= 0.25). (However, the interaction term does not detect departures from additivity.)

**Table 5:** Adjusted odds ratios for breast cancer and cleaning product use stratified by disease causation beliefs

Beliefs about environmental chemicals/pollutants and breast cancer										
	**Contributes "a lot"**					**Does not contribute "a lot"**				

**Product category**	**Cases (no.)**	**Controls (no.)**	**Adj. OR**	**95% CI**	***P ***_**trend**_	**Cases (no.)**	**Controls (no.)**	**Adj. OR**	**95% CI**	***P ***_**trend**_

Combined cleaning product use										

Quartile 1	39	55	1.0	Ref.		52	44	1.0	Ref.	

Quartile 2	58	69	1.4	0.8, 2.4		42	38	0.9	0.5, 1.8	

Quartile 3	71	74	1.6	0.9, 2.8		41	51	0.8	0.4, 1.4	

Quartile 4	77	47	3.2	1.8, 5.9	0.0001	27	23	1.2	0.6, 2.6	0.96

										

Combined air freshener use (sprays and solids)										

Quartile 1	34	43	1.0	Ref.		40	34	1.0	Ref.	

Quartile 2	67	71	1.3	0.7, 2.4		46	46	0.9	0.5, 1.7	

Quartile 3	76	86	1.3	0.7, 2.2		47	52	0.8	0.4, 1.6	

Quartile 4	69	46	2.4	1.3, 4.5	0.01	32	25	1.4	0.7, 3.0	0.53

										

Air freshener spray										

Never use	44	50	1.0	Ref.		46	45	1.0	Ref.	

Any use	203	196	1.3	0.8, 2.1		119	112	1.2	0.7, 2.0	

										

< Once a month	50	57	1.1	0.6, 2.0		33	31	1.1	0.6, 2.2	

Monthly	32	32	1.2	0.6, 2.3		15	9	1.9	0.7, 5.0	

Weekly	71	62	1.5	0.8, 2.6		43	48	1.0	0.6, 2.0	

Daily	50	45	1.4	0.8, 2.7	0.12	28	24	1.2	0.6, 2.6	0.66

										

Solid air freshener										

Never use	144	174	1.0	Ref.		115	114	1.0	Ref.	

Any use	102	72	1.9	1.3, 2.9		51	43	1.4	0.8, 2.3	

										

< 2/year	27	28	1.3	0.7, 2.3		23	13	1.9	0.9, 4.1	

2-6/year	58	32	2.6	1.6, 4.4		19	26	0.9	0.4, 1.8	

≥ 7/year	17	12	1.7	0.8, 3.9	0.0007	9	4	2.8	0.8, 10.2	0.31

										

Oven cleaner										

Never use	11	19	1.0	Ref.		22	14	1.0	Ref.	

Any use	236	227	1.8	0.8, 4.0		143	143	0.6	0.3, 1.2	

										

< 2/year	96	86	2.0	0.9, 4.6		49	57	0.4	0.1, 1.3	

2-6/year	112	121	1.5	0.6, 34		87	75	0.7	0.3, 1.5	

≥ 7/year	28	20	2.4	0.9, 6.5	0.58	7	11	0.4	0.1, 1.3	0.73

										

Surface cleaner										

Never use	29	36	1.0	Ref.		24	18	1.0	Ref.	

Any use	218	209	1.5	0.9,2.7		141	139	0.7	0.4,1.5	

										

< Once a month	23	30	0.9	0.4, 1.9		38	30	0.9	0.4, 2.0	

Monthly	39	36	1.5	0.7, 3.1		18	21	0.6	0.2, 1.4	

Weekly	120	103	1.7	1.0, 3.0		66	68	0.7	0.3, 1.5	

Daily	36	40	1.7	0.8, 3.6	0.02	19	20	0.8	0.3, 2.1	0.45

										

Mold/mildew control										

Never use	166	197	1.0	Ref.		130	125	1.0	Ref.	

Any use	80	49	2.1	1.4, 3.3		34	32	1.1	0.6, 2.0	

										

Mold/mildew control with bleach										

Never use	179	202	1.0	Ref.		141	132	1.0	Ref.	

Any use	67	44	1.8	1.2, 2.9		23	24	1.0	0.5, 2.0	

										

< Once a month	33	25	1.4	0.8, 2.5		14	13	1.1	0.5, 2.4	

Monthly	10	7	1.8	0.6, 5.1		4	4	1.1	0.3, 4.7	

≥ Weekly	24	12	3.2	1.4, 7.1	0.002	5	7	0.8	0.2, 2.7	0.83

Odds ratios are adjusted for age at diagnosis/reference year, birth decade (six categories), previous breast cancer diagnosis, family history of breast cancer, age at first live or still birth (< 30, ≥ 30/nulliparous), education (five categories). "Combined cleaning product use" product category combines frequency of use across five product categories: air freshener spray, solid air freshener, oven cleaner, surface cleaner, and mold/mildew control with bleach.

Similarly, odds ratios for pesticides were higher among participants who believed that chemicals/pollutants contribute "a lot" to breast cancer. For example, the odds ratio for most frequent insect repellent use was 2.0 (95% CI: 1.1, 3.4) in this belief group compared with 0.8 (95% CI: 0.4, 1.6) among others. Pesticide odds ratios stratified by beliefs are shown in Table 6.

**Table 6:** Adjusted odds ratios for breast cancer and residential pesticide use stratified by disease causation beliefs

Beliefs about environmental chemicals/pollutants and breast cancer										
	**Contributes "a lot"**					**Does not contribute "a lot"**				

**Product category**	**Cases (no.)**	**Controls (no.)**	**Adj. OR**	**95% CI**	**P **_**trend**_	**Cases (no.)**	**Controls (no.)**	**Adj. OR**	**95% CI**	**P **_**trend**_

Combined pesticide use										

Quartile 1	91	87	1.0	Ref.		82	65	1.0	Ref.	

Quartile 2	66	47	1.5	0.9, 2.5		44	52	0.7	0.4, 1.1	

Quartile 3	104	89	1.2	0.8, 1.9		65	54	1.0	0.6, 1.7	

Quartile 4	106	75	1.5	1.0, 2.4	0.16	47	51	0.7	0.4, 1.3	0.53

										

Insect or bug control										

Never use	81	78	1.0	Ref.		80	73	1.0	Ref.	

Any use	367	305	1.2	0.9, 1.8		202	209	0.9	0.6, 1.3	

										

Once or twice	105	90	1.1	0.7, 1.8		56	65	0.8	0.5, 1.3	

3-10 times	130	117	1.1	0.8, 1.7		73	71	1.0	0.6, 1.6	

> 10 times	132	98	1.4	0.9, 2.1	0.12	73	73	0.9	0.6, 1.4	0.86

										

Termites/carpenter ants										

Never use	161	146	1.0	Ref		132	119	1.0	Ref	

Any use	112	102	1.0	0.7, 1.4		53	59	0.7	0.4, 1.1	

										

Once or twice	68	54	1.1	0.7, 1.7		37	31	1.0	0.5, 1.7	

3-10 times	28	30	0.9	0.5, 1.6		7	19	0.2	0.1, 0.6	

> 10 times	16	18	0.8	0.4, 1.7	0.55	9	9	0.7	0.3, 2.1	0.06

										

Mosquito control										

Never use	176	186	1.0	Ref.		138	126	1.0	Ref.	

Any use	65	58	1.1	0.7, 1.7		26	29	0.8	0.4, 1.4	

										

Once or twice	10	11	1.2	0.7, 2.2		5	7	0.7	0.2, 2.3	

3-10 times	23	22	1.1	0.6, 2.1		12	9	1.2	0.5, 3.2	

> 10 times	32	25	1.2	0.7, 2.2	0.47	9	13	0.5	0.2, 1.4	0.33

										

Mothball control										

Never use	40	56	1.0	Ref.		33	35	1.0	Ref.	

Any use	207	190	1.3	0.8, 2.1		133	122	1.0	0.6,1.8	

										

< 5 times	50	55	1.2	0.7, 2.1		42	35	1.3	0.7, 2.7	

5-10 times	40	53	1.0	0.5, 1.8		22	20	0.9	0.4, 2.0	

> 10 times	117	82	1.6	1.0, 2.8	0.06	69	67	0.9	0.5, 1.7	0.41

										

Lawn care										

Never use	190	169	1.0	Ref.		126	117	1.0	Ref.	

Any use	250	196	1.1	0.8,1.5		158	147	1.1	0.8,1.5	

										

Once or twice	24	21	1.0	0.5, 2.0		19	14	1.4	0.7, 3.0	

3-20 times	115	83	1.2	0.8, 1.7		59	53	1.1	0.7, 1.8	

> 20 times	111	92	1.0	0.7, 1.5	0.58	80	80	1.0	0.6, 1.5	0.98

										

Outdoor and indoor plant care										

Never use	235	198	1.0	Ref.		172	161	1.0	Ref.	

Any use	214	173	1.0	0.8, 1.4		120	127	0.8	0.6, 1.2	

										

Once or twice	18	12	1.2	0.5, 2.6		15	14	0.9	0.4, 2.0	

3-20 times	104	86	1.0	0.7, 1.5		54	60	0.8	0.5, 1.2	

> 20 times	92	75	1.0	0.7, 1.4	0.99	51	53	0.9	0.5, 1.4	0.39

										

Insect repellent										

Never use	153	134	1.0	Ref.		133	137	1.0	Ref.	

Any use	312	261	1.2	0.9, 1.6		170	167	1.2	0.8, 1.7	

										

Rarely	179	149	1.2	0.8, 1.6		104	114	1.1	0.7, 1.6	

Sometimes	85	85	1.0	0.6, 1.5		48	30	1.9	1.1, 3.4	

Often/Very often	48	27	2.0	1.1, 3.4	0.12	18	23	0.8	0.4, 1.6	0.45

										

Lice control										

Never use	414	344	1.0	Ref.		278	282	1.0	Ref.	

Any use	59	58	1.1	0.7, 1.7		30	25	1.4	0.8, 2.5	

										

Flea collar for pets										

No	132	122	1.0	Ref.		125	116	1.0	Ref.	

Yes	344	290	1.3	0.9, 1.8		185	192	1.0	0.7, 1.4	

										

Flea control for pets										

Never use	256	214	1.0	Ref.		209	181	1.0	Ref.	

Any use	196	177	1.1	0.8, 1.4		98	109	0.8	0.5,1.1	

										

Once or twice	23	23	0.9	0.5, 1.6		20	18	1.0	0.5, 2.1	

3-10 times	63	74	0.8	0.5, 1.2		38	35	0.9	0.6, 1.6	

> 10 times	110	80	1.4	0.9, 2.0	0.27	40	56	0.6	0.4, 1.0	0.07

Odds ratios are adjusted for age at diagnosis/reference year, birth decade (six categories), previous breast cancer diagnosis, family history of breast cancer, age at first live or still birth (< 30, ≥ 30/nulliparous), education (five categories), study (Cape, PCE). "Combined pesticide use" product category includes frequency data for: insect or bug control, lawn care, outdoor and indoor plant care, insect repellent, flea control on pets. Product use for termite or carpenter ant control, mosquito control, and mothball control not included because they were only assessed in study participants from the 1999-2000 interviews.

**Figure 1:**
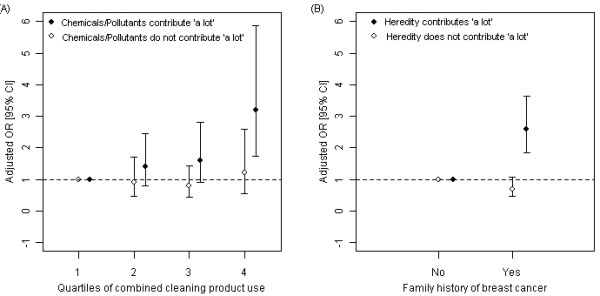
**Cleaning product use, family history, and risk of breast cancer, stratified by beliefs about causation**. Adjusted odds ratios are shown for breast cancer and A) combined cleaning product use stratified by beliefs about environmental chemicals and breast cancer and B) family history of breast cancer stratified by beliefs about heredity and breast cancer, among participants living in Cape Cod, Massachusetts, 1988-1995. Odds ratios are adjusted for age, previous breast cancer diagnosis, age at first birth, and education; additionally, Figure 1A is adjusted for family history of breast cancer and Figure 1B is adjusted for study.

In addition, a similar pattern was observed in the odds ratios for family history of breast cancer stratified by beliefs about heredity as a cause. The odds ratio for breast cancer and family history was markedly higher among women who believed that heredity contributes "a lot" (OR = 2.6, 95% CI: 1.9, 3.6) and not elevated among others (OR = 0.7, 95% CI: 0.5, 1.1, interaction term *P *< 0.01). The parallel pattern of results for both cleaning products and family history when stratified by relevant beliefs is shown in Figure 1. (For all participants, the odds ratio for family history was 1.4 (95% CI: 1.1, 1.9)). The un-stratified and stratified effect estimates for family history of breast cancer in adjusted models remain virtually unchanged after removing subjects with imputed values for family history.

## Discussion

Women with the highest combined cleaning product use had two-fold increased breast cancer risk compared to those with the lowest reported use. Use of air fresheners and products for mold and mildew control were associated with increased risk. To our knowledge, this is the first published report on cleaning product use and risk of breast cancer.

Some common ingredients of air fresheners and products for mold and mildew have been identified as EDCs or carcinogens, supporting the biological plausibility of the elevated odds ratios we observed [1,15,41-51]. EDCs such as synthetic musks and phthalates are commonly used in air fresheners [19,25-27,43,48,52-54] and antimicrobials, phthalates, and alkylphenolic surfactants are often in mold and mildew products [19,22-24,26,27,41,42,44,47,49,55]. In addition, air fresheners may contain: terpenes, which can react with background ozone to form formaldehyde, a human carcinogen [50]; benzene and styrene [51], which are animal mammary gland carcinogens [1]; and other chemicals whose mechanisms of action are not understood [56]. Although exposure levels may be low and EDCs are typically less potent than endogenous hormones, limited knowledge of product formulations, exposure levels, and the biological activity and toxicity of chemical constituents alone and in combination make it difficult to assess risks associated with product use. Additionally, the products we assessed may be proxies for other products that we did not include, and mold/mildew products may be proxies for exposure to mycotoxins, some of which are EDCs [2,57-59].

Our results do not corroborate the findings of a Long Island, NY, case-control study [31]. The Long Island study found increased breast cancer risk associated with self-reported overall pesticide use and use of lawn and garden pesticides, but we did not. Neither study found associations for nuisance pest control (roaches, ants, etc.). While we observed increased risk with frequent use of insect repellent, the Long Island study did not. Differences between the studies may be due to differences in pesticide practices in the two regions, greater statistical power in the Long Island study, or differences in the survey instruments. Phthalates and permethrins, which are in some insect repellents, have been identified as EDCs [10,13,46,60].

Using interviews to assess product-related exposures, as we did in this study, has several advantages. It is inexpensive, noninvasive, and integrates exposures over many years and to frequently-occurring chemical mixtures. Currently available biological measures cannot achieve these important characteristics.

However, self-reported exposures are subject to multiple sources of error resulting in misclassification. Our questions were cognitively demanding in that they asked participants to report behaviors occurring months to years before. Responses failed to capture use by others, including residues from before the participant moved into the residence; exposures specific to critical periods such as adolescence; exposures outside the home; or all products that contain the chemicals of interest. Although we asked about the first and most recent years of pesticide use, we considered the quality of these data inadequate to evaluate effects of duration of use. Much of the error resulting from limitations in exposure measurement is likely nondifferential, biasing odds ratios toward the null.

Self-reports are also vulnerable to bias from differential recall between cases and controls. Women diagnosed with breast cancer may have searched their history for explanations, priming greater recall of product use than for controls. Werler [39], among others, hypothesizes that this type of bias occurs when cases are aware of the study hypothesis, resulting in higher exposure reporting and, consequently, an elevated odds ratio. We empirically investigated this possibility by stratifying odds ratios by beliefs about breast cancer causes, and, consistent with Werler's hypothesis, we observed higher odds ratios for product use among women who believe chemicals and pollution contribute "a lot" to breast cancer than among others.

However, the family history odds ratios stratified by beliefs suggest another interpretation. The much higher family history odds ratios for women who said heredity contributes "a lot" is unlikely to be primarily due to recall bias, given that self-reporting of first degree family members with breast cancer is generally accurate [61-66]. Previous research indicates that over-reporting of first degree breast cancer family history is negligible [63,65,66] and that some under-reporting by controls in comparison with cases is likely to occur (and could bias odds ratios), but this effect is unlikely to be substantial [64-66]. More likely, our results are primarily driven by cases who formed their belief that heredity does not contribute "a lot" after their own diagnosis, based on their own lack of relatives with breast cancer. Our data support this idea: 36% of cases with no family history said heredity contributes "a lot" to breast cancer compared with 61% of cases who did have a family history (Table 7). In this situation, an odds ratio for women who do not think heredity contributes "a lot" over-represents cases with no family history, lowering the effect estimate. Thus, our results support Weiss's argument [40] that limiting estimates to a subgroup based on beliefs about disease causation may introduce error. Among the group who do not believe heredity contributes "a lot" to breast cancer, the odds ratio of 0.7 (95% CI: 0.5, 1.1) contrasts sharply with the pooled odds ratio of 2.1 (95% CI: 2.0, 2.2) for first degree family history of breast cancer from previous studies [67]. Generally, Weiss argues, effect estimates based on one belief or knowledge subgroup lack precision and may underestimate the true effect, since they are limited to smaller numbers and not representative of the study population [40].

**Table 7:** Beliefs about heredity as a cause of breast cancer by family history and case status

		Cases				Controls			
		**Family history of breast cancer**				**Family history of breast cancer**			

		**Yes**		**No**		**Yes**		**No**	

**Belief**		**N**	**%**	**N**	**%**	**N**	**%**	**N**	**%**

Heredity contributes "a lot" to breast cancer	Yes	120	61	211	36	83	61	391	67
	
	No	76	39	380	64	52	39	195	33

The divergent odds ratios in the stratified analysis for family history, which is not likely affected much by recall bias, warns us that the elevated odds ratios for cleaning products should not be too quickly dismissed as resulting from recall bias, since an alternative interpretation is that women's beliefs about disease causation result from their experience. Women who have been intensive product users and are then diagnosed with breast cancer may form the belief that chemicals influenced their risk, or they may be sensitized to news media stories about associations between chemicals and disease and form beliefs from this experience. Social scientists have studied the phenomenon of health beliefs formed from experience in a variety of settings, including the emergence of beliefs about environmental causation among breast cancer activists [68].

Furthermore, the substantial underestimate of risk for family history among women who said heredity does not contribute "a lot" cautions us against limiting product use analyses to a non-belief subgroup as a strategy for dealing with possible recall bias. In addition, the findings of elevated risk for some cleaning products and not others lends evidence that recall bias may not account for elevated risks, even if it contributes in part, since bias would be expected to similarly influence reporting for all the products.

Studies that rely on questionnaire data can sometimes assess the validity of self-reported data against another metric, such as chemical concentrations in relevant exposure media. For example, Colt et al. [69] found significant associations between self-reports of type of pest treated and concentrations of specific pesticides in house dust. We collected air, dust, and urine measurements for 120 homes and their residents, but comparison of these data with self-reports was not conducted for several reasons. The number of homes is small, the one-time environmental measurements may not correspond well with product use over years, measurements capture sources other than home product use, and our self-reports cover past residences as well as the sampled homes. Our ambiguous self-report findings point to the value of thoughtfully incorporating environmental chemical measurements into prospective cohort studies such as the National Children's Study and the Sister Study.

Overall strengths of our study are the population-based design with case identification from the MCR, extensive interviews allowing evaluation of possible confounding by established and hypothesized breast cancer risk factors, and assessment of exposures that extend years before diagnosis and encompass chemicals in use during the past 30 years as well as the more-studied banned organochlorines. Limitations include loss of information due to deaths of women with less treatable cancers. Also, we lack a truly unexposed reference group, limiting contrast in levels of exposure. The self-reported product use exposures have potential for differential and nondifferential error. We did not have adequate numbers to separately evaluate effects in younger women, though some other studies suggest that environmental pollutants may have greater influence on premenopausal disease [28].

To our knowledge, this is the first epidemiological study to suggest an association between cleaning product use, in particular air fresheners and products for mold and mildew control, and elevated breast cancer risk. This association is biologically plausible based on ingredients of these products, such as musks, antimicrobials, and phthalates [1-27,41-49,70-73], and these reported exposures may be proxies for other un-assessed causative exposures. The modest association and possibility of recall bias make interpretation tentative. Given widespread exposure to cleaning products and scented products, follow-up study is important. Prospective designs, which avoid differential recall, can be helpful. The difficulty of obtaining human evidence on environmental chemicals and breast cancer in the short-term means we must rely more on laboratory evidence as a basis for public health policies to control exposure.

## Conclusions

Laboratory studies have found that many chemicals in home-use pesticides and household cleaning products are mammary gland carcinogens in rodents, influence the proliferation of estrogen-sensitive cells, or affect mammary gland development following prenatal exposure. These findings suggest effects of pesticide and cleaning product use on breast cancer risk, so we undertook a case-control study of breast cancer and self-reported product use. We found increased breast cancer risk among women reporting the highest use of cleaning products and air fresheners. We found little association with home pesticide use. The self-reported product use measures we used have the advantage of integrating exposure over many years to chemical mixtures. However, these measures remain incomplete, likely resulting in nondifferential misclassification, and they are open to recall bias. Investigators sometimes try to avoid the influence of recall bias by limiting analyses to participants who do not subscribe to the study hypothesis, but our results show this may not be a good strategy, given that in our study it would obscure the well-established association between family history and breast cancer risk. In order to avoid possible recall bias, we recommend further study of cleaning products and breast cancer using prospective self-reports and measurements in environmental and biological media.

## Abbreviations

CI: confidence interval; CMS: Centers for Medicare and Medicaid Services; EDCs: endocrine-disrupting compounds; OR: odds ratio; MCR: Massachusetts Cancer Registry; PCE: tetrachloroethylene; Ref: reference; Adj OR: adjusted odds ratio; NY: New York; US: United States.

## Competing interests

The authors declare that they have no competing interests.

## Authors' contributions

ARZ conducted the statistical analyses and led drafting of the manuscript. AA designed and oversaw the PCE Study; contributed to the design, data collection, and epidemiological analysis of the Cape Cod Study; and collaborated on editorial issues. RAR contributed to the design, data collection, and analysis of the Cape Cod Study, particularly with respect to the toxicologic characteristics of exposures, and collaborated in drafting the manuscript. JGB led the design, implementation, and analysis of the Cape Cod Study and collaborated in drafting the manuscript; she conceptualized the comparative analysis of product use and family history odds ratios stratified by beliefs as a strategy for understanding possible response bias. All authors read and approved the final manuscript.

## References

[B1] Rudel RA Attfield KR Schifano J Brody JG Chemicals causing mammary gland tumors in animals signal new directions for epidemiology, chemicals testing, and risk assessment for breast cancer prevention Cancer 2007 109 2635 2666 10.1002/cncr.22653 17503434

[B2] Soto AM Sonnenschein C Chung KL Fernandez MF Olea N Serrano FO The E-SCREEN assay as a tool to identify estrogens: an update on estrogenic environmental pollutants Environ Health Perspect 1995 103 Suppl 7 113 122 10.2307/3432519 8593856 PMC1518887

[B3] Fenton SE Endocrine-disrupting compounds and mammary gland development: early exposure and later life consequences Endocrinology 2006 147 S18 24 10.1210/en.2005-1131 16690811

[B4] Sibinski LJ Two year oral (diet) toxicity/carcinogenicity study of fluorochemical FC-143 in rats 3M Company/Riker Exp No 0281CR0012 1987 3M Company/Riker

[B5] National Toxicology Program Abstracts of NTP long-term cancer studies 2007 Research Triangle Park, NC: National Institute of Environmental Health Sciences

[B6] Uzumcu M Kuhn PE Marano JE Armenti AE Passantino L Early postnatal methoxychlor exposure inhibits folliculogenesis and stimulates anti-Mullerian hormone production in the rat ovary J Endocrinol 2006 191 549 558 10.1677/joe.1.06592 17170213

[B7] Morinaga H Yanase T Nomura M Okabe T Goto K Harada N Nawata H A benzimidazole fungicide, benomyl, and its metabolite, carbendazim, induce aromatase activity in a human ovarian granulose-like tumor cell line (KGN) Endocrinology 2004 145 1860 1869 10.1210/en.2003-1182 14691014

[B8] Maranghi F Rescia M Macri C Di Consiglio E De Angelis G Testai E Farini D De Felici M Lorenzetti S Mantovani A Lindane may modulate the female reproductive development through the interaction with ER-beta: an in vivo-in vitro approach Chem Biol Interact 2007 169 1 14 10.1016/j.cbi.2007.04.008 17537412

[B9] Liu P Song XX Yuan WH Wen WH Wu XN Li J Chen XM Effects of cypermethrin and methyl parathion mixtures on hormone levels and immune functions in Wistar rats Archives of Toxicology 2006 80 449 457 10.1007/s00204-006-0071-7 16496128

[B10] Jin Y Chen R Sun L Wang W Zhou L Liu W Fu Z Enantioselective induction of estrogen-responsive gene expression by permethrin enantiomers in embryo-larval zebrafish Chemosphere 2009 74 1238 1244 10.1016/j.chemosphere.2008.11.015 19095286

[B11] Gwinn MR Whipkey DL Tennant LB Weston A Differential gene expression in normal human mammary epithelial cells treated with malathion monitored by DNA microarrays Environ Health Perspect 2005 113 1046 1051 10.1289/ehp.7311 16079077 PMC1280347

[B12] Cupp AS Skinner MK Actions of the endocrine disruptor methoxychlor and its estrogenic metabolite on in vitro embryonic rat seminiferous cord formation and perinatal testis growth Reprod Toxicol 2001 15 317 326 10.1016/S0890-6238(01)00124-1 11390175

[B13] Chen HY Xiao JG Hu G Zhou JW Xiao H Wang XR Estrogenicity of organophosphorus and pyrethroid pesticides Journal of Toxicology and Environmental Health-Part A 2002 65 1419 1435 10.1080/00984100290071243 12396874

[B14] Enoch RR Stanko JP Greiner SN Youngblood GL Rayner JL Fenton SE Mammary gland development as a sensitive end point after acute prenatal exposure to an atrazine metabolite mixture in female Long-Evans rats Environ Health Perspect 2007 115 541 547 10.1289/ehp.9612 17450222 PMC1852649

[B15] Moon HJ Han SY Shin JH Kang IH Kim TS Hong JH Kim SH Fenton SE Gestational exposure to nonylphenol causes precocious mammary gland development in female rat offspring J Reprod Dev 2007 53 333 344 10.1262/jrd.18055 17190974

[B16] Vorderstrasse BA Fenton SE Bohn AA Cundiff JA Lawrence BP A novel effect of dioxin: exposure during pregnancy severely impairs mammary gland differentiation Toxicol Sci 2004 78 248 257 10.1093/toxsci/kfh062 14718648

[B17] White SS Kato K Jia LT Basden BJ Calafat AM Hines EP Stanko JP Wolf CJ Abbott BD Fenton SE Effects of perfluorooctanoic acid on mouse mammary gland development and differentiation resulting from cross-foster and restricted gestational exposures Reprod Toxicol 2009 27 289 298 10.1016/j.reprotox.2008.11.054 19095057 PMC3477546

[B18] Vandenberg LN Maffini MV Schaeberle CM Ucci AA Sonnenschein C Rubin BS Soto AM Perinatal exposure to the xenoestrogen bisphenol-A induces mammary intraductal hyperplasias in adult CD-1 mice Reprod Toxicol 2008 26 210 219 10.1016/j.reprotox.2008.09.015 18938238 PMC3922631

[B19] Centers for Disease Control and Prevention Third national report on human exposure to environmental chemicals National Center for Environmental Health, Division of Laboratory Science 2005

[B20] Calafat AM Kuklenyik Z Reidy JA Caudill SP Tully JS Needham LL Serum concentrations of 11 polyfluoroalkyl compounds in the u.s. population: data from the national health and nutrition examination survey (NHANES) Environ Sci Technol 2007 41 2237 2242 10.1021/es062686m 17438769

[B21] Calafat AM Wong LY Ye X Reidy JA Needham LL Concentrations of the sunscreen agent benzophenone-3 in residents of the United States: National Health and Nutrition Examination Survey 2003--2004 Environ Health Perspect 2008 116 893 897 10.1289/ehp.11269 18629311 PMC2453157

[B22] Calafat AM Ye X Wong LY Reidy JA Needham LL Urinary concentrations of triclosan in the U.S. population: 2003-2004 Environ Health Perspect 2008 116 303 307 10.1289/ehp.10768 18335095 PMC2265044

[B23] Calafat AM Ye X Wong LY Reidy JA Needham LL Exposure of the U.S. population to bisphenol A and 4-tertiary-octylphenol: 2003-2004 Environ Health Perspect 2008 116 39 44 10.1289/ehp.10753 18197297 PMC2199288

[B24] Calafat AM Kuklenyik Z Reidy JA Caudill SP Ekong J Needham LL Urinary concentrations of bisphenol A and 4-nonylphenol in a human reference population Environ Health Perspect 2005 113 391 395 10.1289/ehp.7534 15811827 PMC1278476

[B25] Kuklenyik Z Bryant XA Needham LL Calafat AM SPE/SPME-GC/MS approach for measuring musk compounds in serum and breast milk J Chromatogr B Analyt Technol Biomed Life Sci 2007 858 177 183 10.1016/j.jchromb.2007.08.027 17870677

[B26] Silva MJ Barr DB Reidy JA Malek NA Hodge CC Caudill SP Brock JW Needham LL Calafat AM Urinary levels of seven phthalate metabolites in the U.S. population from the National Health and Nutrition Examination Survey (NHANES) 1999-2000 Environ Health Perspect 2004 112 331 338 14998749 10.1289/ehp.6723 PMC1241863

[B27] Rudel RA Camann DE Spengler JD Korn LR Brody JG Phthalates, alkylphenols, pesticides, polybrominated diphenyl ethers, and other endocrine-disrupting compounds in indoor air and dust Environmental Science & Technology 2003 37 4543 4553 10.1021/es0264596 14594359

[B28] Brody JG Moysich KB Humblet O Attfield KR Beehler GP Rudel RA Environmental pollutants and breast cancer: epidemiologic studies Cancer 2007 109 2667 2711 10.1002/cncr.22655 17503436

[B29] Snedeker SM Pesticides and breast cancer risk: A review of DDT, DDE, and Dieldrin Environmental Health Perspectives 2001 109 35 47 10.2307/3434845 11250804 PMC1240541

[B30] Cohn BA Wolff MS Cirillo PM Sholtz RI DDT and Breast Cancer in Young Women: New Data on the Significance of Age at Exposure Environmental Health Perspectives 2007 115 1406 1414 17938728 10.1289/ehp.10260 PMC2022666

[B31] Teitelbaum SL Gammon MD Britton JA Neugut AI Levin B Stellman SD Reported residential pesticide use and breast cancer risk on Long Island, New York American Journal of Epidemiology 2007 165 643 651 10.1093/aje/kwk046 17166928

[B32] Coughlin SS Recall bias in epidemiologic studies J Clin Epidemiol 1990 43 87 91 10.1016/0895-4356(90)90060-3 2319285

[B33] State Cancer Profiles http://statecancerprofiles.cancer.gov/map/scpMapDataTable.php?25&001&055&00&2&1&0&1&6&0

[B34] Cape Cod Breast Cancer and the Environment Atlas http://library.silentspring.org/atlas/breastcancer/index.asp

[B35] Silent Spring Institute Cape Cod Breast Cancer and Environment Study: Final Report 1997 Newton, MA

[B36] McKelvey W Brody JG Aschengrau A Swartz CH Association between residence on Cape Cod, Massachusetts, and breast cancer Ann Epidemiol 2004 14 89 94 10.1016/S1047-2797(03)00120-0 15018880

[B37] Brody JG Aschengrau A McKelvey W Rudel RA Swartz CH Kennedy T Breast cancer risk and historical exposure to pesticides from wide-area applications assessed with GIS Environ Health Perspect 2004 112 889 897 15175178 10.1289/ehp.6845 PMC1242018

[B38] Aschengrau A Rogers S Ozonoff D Perchloroethylene-contaminated drinking water and the risk of breast cancer: additional results from Cape Cod, Massachusetts, USA Environmental Health Perspectives 2003 111 167 173 12573900 10.1289/ehp.4980 PMC1241345

[B39] Werler MM Shapiro S Mitchell AA Periconceptional folic acid exposure and risk of occurrent neural tube defects Jama 1993 269 1257 1261 10.1001/jama.269.10.1257 8437302

[B40] Weiss NS Should we consider a subject's knowledge of the etiologic hypothesis in the analysis of case-control studies? Am J Epidemiol 1994 139 247 249 8116599 10.1093/oxfordjournals.aje.a116991

[B41] Ahn KC Zhao B Chen J Cherednichenko G Sanmarti E Denison MS Lasley B Pessah IN Kultz D Chang DP In vitro biologic activities of the antimicrobials triclocarban, its analogs, and triclosan in bioassay screens: receptor-based bioassay screens Environ Health Perspect 2008 116 1203 1210 10.1289/ehp.11200 18795164 PMC2535623

[B42] Bonefeld-Jorgensen EC Long M Hofmeister MV Vinggaard AM Endocrine-disrupting potential of bisphenol A, bisphenol A dimethacrylate, 4-n-nonylphenol, and 4-n-octylphenol in vitro: new data and a brief review Environmental Health Perspectives 2007 115 Suppl 1 69 76 10.1289/ehp.9368 18174953 PMC2174402

[B43] Duty SM Ackerman RM Calafat AM Hauser R Personal care product use predicts urinary concentrations of some phthalate monoesters Environmental Health Perspectectives 2005 113 1530 1535 10.1289/ehp.8083 PMC1310914 16263507

[B44] Gee RH Charles A Taylor N Darbre PD Oestrogenic and androgenic activity of triclosan in breast cancer cells J Appl Toxicol 2008 28 78 91 10.1002/jat.1316 17992702

[B45] Hauser R Meeker JD Duty S Silva MJ Calafat AM Altered semen quality in relation to urinary concentrations of phthalate monoester and oxidative metabolites Epidemiology 2006 17 682 691 10.1097/01.ede.0000235996.89953.d7 17003688

[B46] Howdeshell KL Wilson VS Furr J Lambright CR Rider CV Blystone CR Hotchkiss AK Gray LE Jr A mixture of five phthalate esters inhibits fetal testicular testosterone production in the sprague-dawley rat in a cumulative, dose-additive manner Toxicol Sci 2008 105 153 165 10.1093/toxsci/kfn077 18411233

[B47] Kumar V Chakraborty A Kural MR Roy P Alteration of testicular steroidogenesis and histopathology of reproductive system in male rats treated with triclosan Reprod Toxicol 2009 27 177 185 10.1016/j.reprotox.2008.12.002 19118620

[B48] Schreurs RH Sonneveld E Jansen JH Seinen W van der Burg B Interaction of polycyclic musks and UV filters with the estrogen receptor (ER), androgen receptor (AR), and progesterone receptor (PR) in reporter gene bioassays Toxicol Sci 2005 83 264 272 10.1093/toxsci/kfi035 15537743

[B49] Zorrilla LM Gibson EK Jeffay SC Crofton KM Setzer WR Cooper RL Stoker TE The effects of triclosan on puberty and thyroid hormones in male Wistar rats Toxicol Sci 2009 107 56 64 10.1093/toxsci/kfn225 18940961

[B50] Nazaroff WW Weschler CJ Cleaning products and air fresheners: exposure to primary and secondary air pollutants Atmospheric Environment 2004 38 2841 2865 10.1016/j.atmosenv.2004.02.040

[B51] Torfs R Brouwere KD Spruyt M Goelen E Nickmilder M Bernard A Exposure and Risk Assessment of Air Fresheners 2008 Flemish Institute for Technological Research NV (VITO) pp. 2008/IMS/R/2222: 2008/IMS/R/2222

[B52] Reiner JL Kannan K A survey of polycyclic musks in selected household commodities from the United States Chemosphere 2006 62 867 873 10.1016/j.chemosphere.2005.10.006 16309730

[B53] Reiner JL Wong CM Arcaro KF Kannan K Synthetic musk fragrances in human milk from the United States Environmental Science & Technology 2007 41 3815 3820 10.1021/es063088a 17612154

[B54] van der Burg B Schreurs R van der Linden S Seinen W Brouwer A Sonneveld E Endocrine effects of polycyclic musks: do we smell a rat? International Journal of Andrology 2008 31 188 193 10.1111/j.1365-2605.2007.00831.x 17971161

[B55] Rudel RA Perovich LJ Endocrine disrupting chemicals in indoor and outdoor air Atmospheric Environment 2009 43 170 181 10.1016/j.atmosenv.2008.09.025 20047015 PMC2677823

[B56] Steinemann AC Fragranced consumer products and undisclosed ingredients Environmental Impact Assessment Review 2009 29 32 38 10.1016/j.eiar.2008.05.002

[B57] Nielsen KF Mycotoxin production by indoor molds Fungal Genetics and Biology 2003 39 103 117 10.1016/S1087-1845(03)00026-4 12781669

[B58] Pestka JJ Yike I Dearborn DG Ward MDW Harkema JR Stachybotrys chartarum, trichothecene mycotoxins, and damp building-related illness: New insights into a public health enigma Toxicological Sciences 2008 104 4 26 10.1093/toxsci/kfm284 18007011

[B59] Tiemann U Tomek W Schneider F Muller M Pohland R Vanselow J The mycotoxins alternariol and alternariol methyl ether negatively affect progesterone synthesis in porcine granulosa cells in vitro Toxicology Letters 2009 186 139 145 10.1016/j.toxlet.2009.01.014 19429235

[B60] Brown M Hebert AA Insect repellents: an overview J Am Acad Dermatol 1997 36 243 249 10.1016/S0190-9622(97)70289-5 9039177

[B61] Chang ET Smedby KE Hjalgrim H Glimelius B Adami HO Reliability of self-reported family history of cancer in a large case-control study of lymphoma J Natl Cancer Inst 2006 98 61 68 10.1093/jnci/djj005 16391372

[B62] Ziogas A Anton-Culver H Validation of family history data in cancer family registries Am J Prev Med 2003 24 190 198 10.1016/S0749-3797(02)00593-7 12568826

[B63] Parent ME Ghadirian P Lacroix A Perret C Accuracy of Reports of Familial Breast-Cancer in a Case-Control Series Epidemiology 1995 6 184 186 10.1097/00001648-199503000-00018 7742408

[B64] Floderus B Barlow L Mack TM Recall bias in subjective reports of familial cancer Epidemiology (Cambridge, Mass) 1990 1 318 321 2083311 10.1097/00001648-199007000-00011

[B65] Murff HJ Spigel DR Syngal S Does this patient have a family history of cancer? An evidence-based analysis of the accuracy of family cancer history JAMA 2004 292 1480 1489 10.1001/jama.292.12.1480 15383520

[B66] Parikh-Patel A Allen M Wright WE Validation of self-reported cancers in the California Teachers Study American journal of epidemiology 2003 157 539 545 10.1093/aje/kwg006 12631544

[B67] Pharoah PD Day NE Duffy S Easton DF Ponder BA Family history and the risk of breast cancer: a systematic review and meta-analysis Int J Cancer 1997 71 800 809 10.1002/(SICI)1097-0215(19970529)71:5<800::AID-IJC18>3.0.CO;2-B 9180149

[B68] Brown P Toxic exposures: contested illnesses and the environmental health movement 2007 New York: Columbia University Press

[B69] Colt JS Lubin J Camann D Davis S Cerhan J Severson RK Cozen W Hartge P Comparison of pesticide levels in carpet dust and self-reported pest treatment practices in four US sites J Expo Anal Environ Epidemiol 2004 14 74 83 10.1038/sj.jea.7500307 14726946

[B70] Kang KS Che JH Ryu DY Kim TW Li GX Lee YS Decreased sperm number and motile activity on the F1 offspring maternally exposed to butyl p-hydroxybenzoic acid (butyl paraben) The Journal of Veterinary Medical Science 2002 64 227 235 10.1292/jvms.64.227 11999442

[B71] Rastogi SC Schouten A de Kruijf N Weijland JW Contents of methyl-, ethyl-, propyl-, butyl- and benzylparaben in cosmetic products Contact dermatitis 1995 32 28 30 10.1111/j.1600-0536.1995.tb00836.x 7720367

[B72] Routledge EJ Parker J Odum J Ashby J Sumpter JP Some alkyl hydroxy benzoate preservatives (parabens) are estrogenic Toxicology and Applied Pharmacology 1998 153 12 19 10.1006/taap.1998.8544 9875295

[B73] Shen HY Jiang HL Mao HL Pan G Zhou L Cao YF Simultaneous determination of seven phthalates and four parabens in cosmetic products using HPLC-DAD and GC-MS methods Journal of Separation Science 2007 30 48 54 10.1002/jssc.200600215 17313141

